# Corneal confocal microscopy for identification of diabetic sensorimotor polyneuropathy: a pooled multinational consortium study

**DOI:** 10.1007/s00125-018-4653-8

**Published:** 2018-06-04

**Authors:** Bruce A. Perkins, Leif E. Lovblom, Vera Bril, Daniel Scarr, Ilia Ostrovski, Andrej Orszag, Katie Edwards, Nicola Pritchard, Anthony Russell, Cirous Dehghani, Danièle Pacaud, Kenneth Romanchuk, Jean K. Mah, Maria Jeziorska, Andrew Marshall, Roni M. Shtein, Rodica Pop-Busui, Stephen I. Lentz, Andrew J. M. Boulton, Mitra Tavakoli, Nathan Efron, Rayaz A. Malik

**Affiliations:** 1 Lunenfeld-Tanenbaum Research Institute, Leadership Sinai Centre for Diabetes, Mount Sinai Hospital, L5209-60 Murray Street, Box 16, Toronto, ON M5T 3L9 Canada; 2 Division of Endocrinology and Metabolism, Department of Medicine, University of Toronto, Toronto, ON Canada; 3 The Ellen and Martin Prosserman Centre for Neuromuscular Diseases, Krembil Neuroscience Centre, Division of Neurology, Department of Medicine, University Health Network, University of Toronto, Toronto, ON Canada; 4 Institute of Health and Biomedical Innovation, Queensland University of Technology, Brisbane, QLD Australia; 5 Faculty of Medicine, University of Queensland, Woolloongabba, QLD Australia; 6 Alberta Children’s Hospital, Department of Paediatrics, Cumming School of Medicine, University of Calgary, Calgary, AB Canada; 7 Department of Surgery, University of Calgary, Calgary, AB Canada; 8 Division of Cardiovascular Sciences, School of Medical Sciences, Faculty of Biology, Medicine and Health, University of Manchester, Manchester, UK; 9 Centre for Endocrinology and Diabetes, School of Medical Sciences, Faculty of Biology, Medicine and Health, University of Manchester, Oxford Road, Manchester, M13 9PL UK; 10 Ophthalmology & Visual Science Department, University of Michigan, Ann Arbor, MI USA; 11 Department of Internal Medicine, Division of Metabolism, Endocrinology & Diabetes, University of Michigan, Ann Arbor, MI USA; 12 Division of Diabetes, Endocrinology & Gastroenterology, School of Medical Sciences, Faculty of Biology, Medicine and Health, University of Manchester, Manchester, UK; 13 Diabetes, Vascular Research Centre, Institute of Health Research, University of Exeter Medical School, Exeter, UK; 14 Department of Medicine, Weill Cornell Medicine-Qatar, Doha, Qatar

**Keywords:** Corneal confocal microscopy, Corneal nerves, Diabetic neuropathy, Diabetic sensorimotor polyneuropathy, Small nerve fibre morphology

## Abstract

**Aims/hypothesis:**

Small cohort studies raise the hypothesis that corneal nerve abnormalities (including corneal nerve fibre length [CNFL]) are valid non-invasive imaging endpoints for diabetic sensorimotor polyneuropathy (DSP). We aimed to establish concurrent validity and diagnostic thresholds in a large cohort of participants with and without DSP.

**Methods:**

Nine hundred and ninety-eight participants from five centres (516 with type 1 diabetes and 482 with type 2 diabetes) underwent CNFL quantification and clinical and electrophysiological examination. AUC and diagnostic thresholds were derived and validated in randomly selected samples using receiver operating characteristic analysis. Sensitivity analyses included latent class models to address the issue of imperfect reference standard.

**Results:**

Type 1 and type 2 diabetes subcohorts had mean age of 42 ± 19 and 62 ± 10 years, diabetes duration 21 ± 15 and 12 ± 9 years and DSP prevalence of 31% and 53%, respectively. Derivation AUC for CNFL was 0.77 in type 1 diabetes (*p* < 0.001) and 0.68 in type 2 diabetes (*p* < 0.001) and was approximately reproduced in validation sets. The optimal threshold for automated CNFL was 12.5 mm/mm^2^ in type 1 diabetes and 12.3 mm/mm^2^ in type 2 diabetes. In the total cohort, a lower threshold value below 8.6 mm/mm^2^ to rule in DSP and an upper value of 15.3 mm/mm^2^ to rule out DSP were associated with 88% specificity and 88% sensitivity.

**Conclusions/interpretation:**

We established the diagnostic validity and common diagnostic thresholds for CNFL in type 1 and type 2 diabetes. Further research must determine to what extent CNFL can be deployed in clinical practice and in clinical trials assessing the efficacy of disease-modifying therapies for DSP.

**Electronic supplementary material:**

The online version of this article (10.1007/s00125-018-4653-8) contains peer-reviewed but unedited supplementary material, which is available to authorised users.

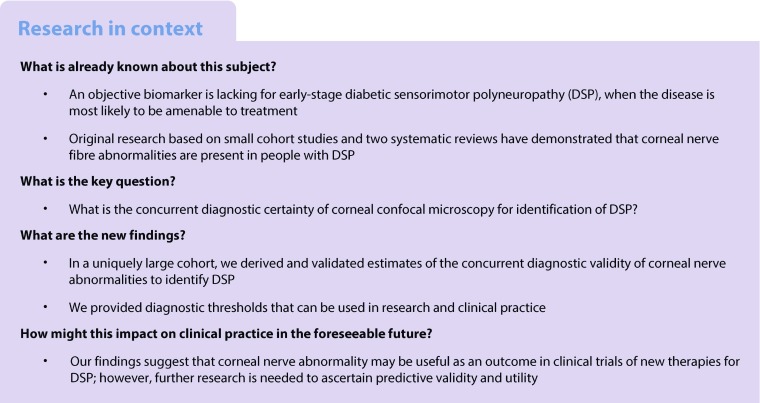



## Introduction

Diabetic sensorimotor polyneuropathy (DSP) occurs in 50–90% of people with diabetes and is a progressive, length-dependent process of nerve injury with complex underlying causal mechanisms [1]. Because of the long subclinical latency period, early identification and management could potentially limit the morbidity and healthcare costs of advanced neuropathy with its associated pain, foot deformity, ulceration and amputation. The diagnosis of DSP is often made late, as neurological and electrophysiological testing of large myelinated fibres identifies established neuropathy [1, 2]. Early identification of unmyelinated small nerve fibre injury will likely provide the best opportunity for effective therapy [1, 3].

Small cohort studies have shown that in vivo corneal confocal microscopy (IVCCM) is an objective and reproducible means to quantify small fibre damage [3]. The rapid non-invasive nature of this procedure and automated image analysis may enable eye specialists to perform this procedure alongside routine examination for diabetic retinopathy [4–7]. However, small cohort studies can be biased in participant selection, in IVCCM image acquisition, in corneal nerve quantification and in defining DSP. We have undertaken a stratified cross-sectional multicentre pooled analysis of 998 participants with diabetes to more definitively establish the diagnostic validity of IVCCM for DSP using manual and automated analysis techniques.

## Methods

### Study population

Five hundred and sixteen people with type 1 diabetes mellitus (432 adults and 84 adolescents) and 482 adults with type 2 diabetes mellitus with and without DSP (total *N* = 998) were studied between 2008 and 2011. Participant-level data was pooled from five separate cohorts as part of a prospective study of diagnostic validity by an international consortium (National Institutes of Health [NIH] grant 1DP3DK104386-01, ClinicalTrials.gov registration no. NCT02423434). Two preliminary reports of diagnostic accuracy from individual centres have previously been published [5, 6]; 190 participants from these two studies are included in this current analysis, representing 19% of this 998-person study. Additional details are provided in electronic supplementary material (ESM) Methods.

### Study design

This is a cross-sectional analysis of baseline visits, reported according to the 2015 Standards for Reporting of Diagnostic Accuracy statement [8]. The diagnostic index test was quantification of corneal nerve morphology obtained by IVCCM, the target condition was DSP, and the reference standard was based on the Toronto consensus criteria incorporating electrophysiological abnormality in the lower limbs [1]. The index test and reference standard were conducted during the same study visit; staff performing the reference standard were blinded to results of the index test (and vice versa). For the index test, participants underwent examination of the sub-basal nerve plexus of the cornea using the Heidelberg Tomograph Rostock Cornea Module III (Heidelberg Engineering GmbH, Heidelberg, Germany and Heidelberg Engineering, Smithfield, RI, USA) according to published methods [9]. Using a manual (MANUAL) and automated (AUTO) protocol [4], corneal nerve fibre length (CNFL), corneal nerve branch density (CNBD) and corneal nerve fibre density (CNFD) were quantified. Published data have demonstrated similar cohort IVCCM characteristics, reproducibility and validity, regardless of study centre. Full details of the index test and reference standard are provided in ESM Methods.

### Statistics

Analysis was stratified by diabetes type and included derivation and validation sets. Baseline characteristics were compared using simple univariable statistics. Receiver operating characteristic (ROC) curves were generated and the AUC, representing diagnostic accuracy, was compared. Optimal diagnostic thresholds were identified by distance to the point of perfect discrimination. Simple random sampling, without replacement with an equal proportion of centre membership, was used to create derivation and validation sets. The following validation criteria were used: (1) validation AUC fell inside the 95% CI of the derivation AUC and (2) the optimal thresholds of the derivation set had similar characteristics to the validation set. The AUC of each test was also compared between subcohorts. An α level of 0.05 was used (two-tailed). Sensitivity analyses were undertaken to account for possible imperfect reference standard and included modification of the reference standard variables to create less- and more-stringent definitions, composite reference standard methods and latent class analysis. ROC regression was used to determine the effects of age and sex on diagnostic accuracy. Alternative diagnostic thresholds were investigated. Additional details of the statistics, and variables used for sensitivity analyses, are provided in ESM Methods.

## Results

Among eligible participants enrolled at the five centres, 516/574 (90%) with type 1 diabetes and 482/527 (91%) with type 2 diabetes underwent the index test and reference standard (ESM Fig. 1). General characteristics of the study population and the diabetes subcohorts are shown in Table 1. DSP was present in 415 (42%) of the study population, in 160 (31%) of the type 1 diabetes subcohort and in 255 (53%) of the type 2 diabetes subcohort. Significantly impaired nerve conduction and IVCCM variables were observed in participants with vs without DSP; the presence of a broad spectrum of neuropathy measures was confirmed (ESM Tables 1 and 2).

**Table 1:** Characteristics of the 998 study participants

Characteristic	Total (*N* = 998)	T1D (*N* = 516)	T2D (*N* = 482)	*p* value for T1D vs T2D
Female sex	420 (42)	255 (49)	165 (34)	<0.001
Age, years	52 ± 18	42 ± 19	62 ± 10	<0.001
Ethnicity				
Aboriginal North American	1 (0)	1 (0)	0 (0)	
Asian	132 (13)	31 (6)	101 (21)	
Black	11 (1)	5 (1)	6 (1)	
Hawaiian or Pacific Islander	1 (0)	0 (0)	1 (0)	
Hispanic	15 (2)	3 (1)	12 (2)	
Middle Eastern	5 (1)	1 (0)	4 (1)	
White	799 (80)	463 (90)	336 (70)	
Other/unknown/unreported	34 (3)	12 (2)	22 (5)	
Diabetes duration, years	17 ± 13	21 ± 15	12 ± 9	<0.001
BMI, kg/m^2^	28.1 ± 6.1	25.6 ± 4.8	31.3 ± 5.9	<0.001
HbA_1c_, mmol/mol	63 ± 18	67 ± 18	59 ± 17	<0.001
HbA_1c_, %	7.9 ± 1.6	8.3 ± 1.6	7.6 ± 1.5	<0.001
Neurological examination				
Sign(s) present	721 (72)	302 (59)	419 (87)	<0.001
Symptom(s) present	606 (61)	211 (41)	395 (82)	<0.001
Nerve conduction studies				
Sural AMP, μV	8.3 ± 7.9	10.2 ± 8.6	6.2 ± 6.4	<0.001
Sural CV, m/s	41.2 ± 7.1	41.4 ± 7.1	41.1 ± 7.2	0.49
Peroneal AMP, mV	3.7 ± 2.6	4.2 ± 2.8	3.2 ± 2.4	<0.001
Peroneal CV, m/s	41.4 ± 7.5	42.0 ± 7.5	40.7 ± 7.3	0.006
Peroneal F wave, ms	57.9 ± 10.3	57.5 ± 9.6	58.3 ± 10.9	0.27
DSP present	415 (42)	160 (31)	255 (53)	<0.001
IVCCM automated protocol variables				
CNFL_AUTO_, mm/mm^2^	12.5 ± 4.6	12.9 ± 4.5	12.2 ± 4.6	0.014
CNBD_AUTO_, branches/mm^2^	22.7 ± 18.3	21.8 ± 16.9	23.7 ± 19.7	0.45
CNFD_AUTO_, fibres/mm^2^	20.6 ± 9.8	20.0 ± 9.4	21.3 ± 10.1	0.043
IVCCM manual protocol variables				
CNFL_MANUAL_, mm/mm^2^	17.3 ± 6.5	17.5 ± 6.2	17.0 ± 6.8	0.21
CNBD_MANUAL_, branches/mm^2^	50.9 ± 40.0	49.6 ± 34.3	52.2 ± 45.4	0.52
CNFD_MANUAL_, fibres/mm^2^	38.6 ± 26.3	31.5 ± 12.0	43.9 ± 32.2	<0.001

Data are presented as mean ± SD or *n* (%)

AMP, amplitude potential; CV, conduction velocity; T1D, type 1 diabetes; T2D, type 2 diabetes

**Fig. 1:**
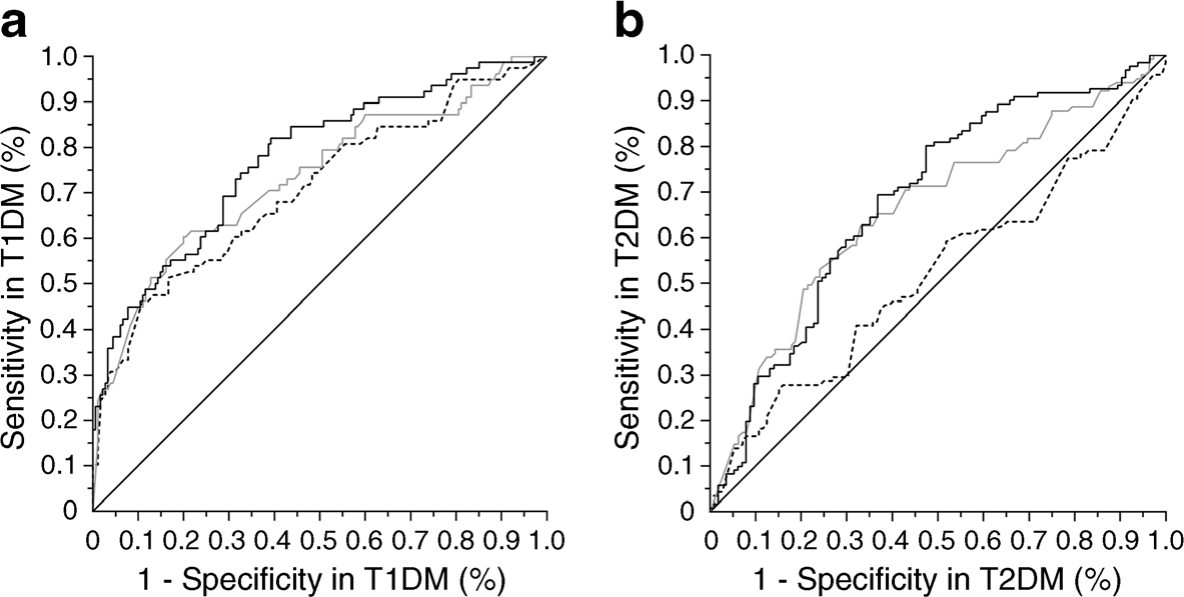
Determination of diagnostic accuracy and optimal thresholds for identification of DSP by IVCCM in the derivation sets. (**a**) Optimal threshold for CNFL_AUTO_ in type 1 diabetes was 12.5 mm/mm^2^, 73% sensitivity and 69% specificity, positive predictive value 50%, negative predictive value 86%, positive likelihood ratio 2.32 and negative likelihood ratio 0.39. (**b**) Optimal threshold for CNFL_AUTO_ in type 2 diabetes was 12.3 mm/mm^2^, 69% sensitivity and 63% specificity, positive predictive value 66%, negative predictive value 66%, positive likelihood ratio 1.86, and negative likelihood ratio 0.49. Continuous black lines, CNFL_AUTO_; grey lines, CNBD_AUTO_; dashed black lines, CNFD_AUTO_. AUC values for CNFL_AUTO_, CNBD_AUTO_ and CNFD_AUTO_ were 0.77, 0.73 and 0.71 in type 1 diabetes, respectively, and 0.68, 0.66 and 0.52 in type 2 diabetes, respectively. The *p* value for comparison of AUC for CNFL_AUTO_ between type 1 and type 2 diabetes derivation sets was not significant at 0.060; when the derivation and validations sets were combined, this *p* value was 0.003. T1DM, type 1 diabetes; T2DM, type 2 diabetes

Fig. 1 displays the ROC curves for IVCCM quantified by the automated protocol in the type 1 diabetes (Fig. 1a) and type 2 diabetes (Fig. 1b) derivation sets. In type 1 diabetes, CNFL_AUTO_ had an AUC of 0.77 and an optimal threshold of 12.5 mm/mm^2^ (73% sensitivity and 69% specificity). In type 2 diabetes, CNFL_AUTO_ had an AUC of 0.68 and an optimal threshold of 12.3 mm/mm^2^ (69% sensitivity and 63% specificity). In both type 1 and type 2 diabetes derivation sets, AUC for CNFL_AUTO_ was significantly greater than 0.50 (which represents a test with no diagnostic accuracy, *p* < 0.001 for both comparisons). CNFL_AUTO_ was associated with the highest AUC among the IVCCM variables in both subcohorts (ESM Table 3). Similar results were observed for IVCCM variables quantified by the manual protocol, and results for all variables were generally confirmed in the validation sets. Full details of the ROC curve analysis are presented in ESM Table 3.

Although the AUC values differed marginally by diabetes type, the optimal thresholds were virtually identical. We thus determined diagnostic accuracy in the full 998-person study (ESM Table 3): CNFL_AUTO_ had an AUC of 0.71 and an optimal threshold of 12.3 mm/mm^2^ (67% sensitivity, 66% specificity, 59% positive predictive value, 74% negative predictive value, 1.97 positive likelihood ratio and 0.50 negative likelihood ratio). CNFL_MANUAL_ had marginally lower AUC (0.70, *p* = 0.006 vs CNFL_AUTO_) but its optimal threshold value of 16.3 mm/mm^2^ had similar operating characteristics. The alternative threshold analysis, in which upper and lower threshold values were used to simultaneously maximise sensitivity and specificity, is shown in ESM Table 4. We noted that in the 998-person group, a lower CNFL_AUTO_ threshold value of <8.6 mm/mm^2^ to rule in DSP and an upper CNFL_AUTO_ threshold value of 15.3 mm/mm^2^ to rule out DSP was associated with 88% specificity and 88% sensitivity.

The sensitivity analyses are summarised in ESM Fig. 2 and ESM Table 5. In type 1 diabetes, more-stringent reference standard definitions resulted in higher AUC for CNFL. Performance using the composite reference test and latent class analysis for DSP case definition resulted in higher AUC (though differences were not statistically significant). No differences were observed in type 2 diabetes. No statistically significant effects of age or sex on ROC curves were found for CNFL.

## Discussion

The findings of this large multicentre pooled concurrent diagnostic validity study reveal that IVCCM had diagnostic validity despite an imperfect reference standard for DSP, using both manual and automated corneal nerve quantification; CNFL was the optimal IVCCM variable and the estimate of performance in the primary analysis was conservative compared with sensitivity analyses that addressed the issue of the imperfect reference standard.

An objective imaging biomarker that can identify early-stage DSP (when interventions are most likely to be effective) and that can be used as an appropriate endpoint in the evaluation of putative therapies does not currently exist [1]. Late diagnosis limits the potential benefits of early risk factor management in preventing neuropathy-related sequelae [2]. The diagnosis of DSP itself is controversial as no definitive gold-standard testing exists aside from electrophysiological evaluation, which primarily identifies later-stage, large-fibre dysfunction and requires considerable specialist expertise, resources and time. In this context, IVCCM represents a rapid, non-invasive imaging endpoint for identifying early small fibre neuropathy. It has been extensively studied in small cross-sectional and cohort studies, which have established normative distributions [10], feasibility, reproducibility and the impact of variations in equipment and procedures.

As electrophysiological testing identifies later-stage rather than early-stage neuropathy, in the current analysis subclinical levels of neuropathy that were not classified as cases might a priori be expected to accentuate false-positives and impair test specificity. We believe this is the major reason for not achieving conventional standards of diagnostic performance and operating characteristics in this study. However, the performance and thresholds are sufficient to raise confidence in automated IVCCM as a diagnostic test [4]. Further research must focus on evaluation of the influence of IVCCM on treatment decisions, possible roles relative to existing tests, its impact on clinical outcomes such as new onset symptomatic neuropathy and foot complications, its role in further evaluation of therapies for neuropathy and its economic impact.

The present study minimised common sources of bias in diagnostic studies, such as recruitment, spectrum and verification bias, but it had limitations. Though common protocols were used, centralised supervision of IVCCM image acquisition and analysis and electrophysiological testing were not implemented. As a cross-sectional analysis, it did not evaluate the predictive validity of IVCCM (a future goal of the consortium). Confirmation of a lack of age effect will require a larger older-adult sample size.

The diagnostic utility of IVCCM has been established in the largest cohort to date and the findings of this study further support the notion that IVCCM is an objective and simple diagnostic test for DSP. Further research must determine to what extent IVCCM can be deployed in clinical practice and in clinical trials assessing the efficacy of disease-modifying therapies for DSP.

## Electronic supplementary material

ESM (PDF 467 kb)

## Data Availability

Data are available from the corresponding author on reasonable request.

## Abbreviations

CNBD: Corneal nerve branch density
CNFD: Corneal nerve fibre density
CNFL: Corneal nerve fibre length
DSP: Diabetic sensorimotor polyneuropathy
IVCCM: In vivo corneal confocal microscopy
ROC: Receiver operating characteristic
