# Supplementary material for: Corneal confocal microscopy for identification of diabetic sensorimotor polyneuropathy: a pooled multinational consortium study
Source: Diabetologia. 2018 Jun 4;61(8):1856–61. doi: 10.1007/s00125-018-4653-8 (PMC6061173; doi:10.1007/s00125-018-4653-8)
Supplement: Supplementary file 1 — (PDF 467 kb) [file 125_2018_4653_MOESM1_ESM.pdf]

ELECTRONIC SUPPLEMENTARY MATERIAL (ESM)

1. ESM METHODS

Study Population

The study population was accrued from the baseline evaluations of a consortium of 5 different cohort studies initiated between 2008 and 2011.[1-5] The objectives of the 5 studies were to determine the role of in vivo corneal confocal microscopy (IVCCM) as a biomarker for diabetic neuropathy. The five study centres included: Queensland University of Technology (Brisbane, QLD, Australia), the University of Calgary (Calgary, AB, Canada), the University of Manchester (Manchester, UK), the University of Michigan (Ann Arbor, MI, USA), and the University of Toronto (Toronto, ON, Canada). Preliminary analyses of diagnostic validity have been published by the Toronto[4] and Manchester,[3] cohorts and the Brisbane site has published other outcomes using baseline data[5]. This current study includes patient-level data from all baseline visits from all five sites, some of which was accrued after the publication of these preliminary works. In September 2014, the NIH funded continued longitudinal follow-up of participants from the studies initiated at each of these sites; this manuscript is the first to be published by this collaboration.

The presence of diabetes mellitus was defined in accordance with American Diabetes Association guidelines. Neuropathy due to non-diabetic causes, current eye infection or other conditions that precluded IVCCM, or allergy to the ocular anesthetic used during the IVCCM exam were exclusions. Neuropathies due to non-diabetic causes were determined through detailed patient history or through screening of immunoglobulins and B12 levels, depending on each site’s local protocol. The protocol and consent procedures at all sites were approved by local research ethics boards, and written informed consent was provided by all study participants or their legal guardians.

Section 1. ESM Methods

Index Test (IVCCM Examination)

Participants underwent examination of the sub-basal nerve plexus of the cornea using the Heidelberg Tomograph Rostock Cornea Module III (Heidelberg Engineering GmbH, Heidelberg, Germany and Heidelberg Engineering, Smithfield, RI, USA) according to published methods.[6] In brief, topical anaesthetic and a viscous gel medium were applied, permitting a visual gel bridge between the cornea and the sterile single-use cap on the microscope's objective lens. Images were taken through the sub-basal layer over a depth of 50 microns using methods that had minor procedural variation between centres.[7] The most technically sound images were identified manually by site staff, and IVCCM parameters were measured using a manual protocol and an automated protocol[8,9] that served as a method of standardization. The latter was also performed as it represents a significant resource-sparing tool that has not been systematically studied for validity compared to the standard manual method. For the manual protocol of image analysis, the examiner traced nerve fibres on the images using a graphic tablet and pen and the parameters were determined using semi-automated analytical software (CCM Image Analysis tool v0.6, developed by M. Dabbah, University of Manchester). For the automated protocol, fully-automated software determined the parameters (ACCMetrics Image Analysis Software v2.0, developed by M. Dabbah and X. Chen, University of Manchester). Results from 1-8 images per eye were averaged. Measured parameters were corneal nerve fibre length (CNFL), expressed as the total length of nerves in mm/mm^2 of image area; corneal nerve branch density (CNBD), expressed as the number of $branches/mm^2$; and corneal nerve fibre density (CNFD), expressed as the number of $fibres/mm^2$. The automated protocol is known to provide measures of CNFL that are systematically 30% lower than manually-derived CNFL.[8,9] Subscripts AUTO and MANUAL indicate automated and manual quantification. Raters were either trained in

optometry or ophthalmology, except Toronto, which used research assistants who underwent two-day training by the microscope manufacturer. Published data have demonstrated similar cohort IVCCM characteristics, reproducibility, and concurrent validity regardless of study centre.[1,2,4,7,10-12]

Reference Standard (Clinical Evaluation and Nerve Conduction Studies)

All study participants underwent nerve conduction studies and comprehensive physical examination. For nerve conduction studies, all investigational sites measured the dominant limb peroneal and sural nerves using clinical nerve conduction study equipment according to the standards of the American Association for Neuromuscular and Electrodiagnostic Medicine. Each centre performed examinations and collected data independently and results were sent to the centre leading statistical analysis (Toronto) where an algorithm was used to determine neuropathy cases. Locally, the results of the neurological examinations were organized into clinical symptom and clinical sign scores, with different centres using different scores according to their baseline study protocols (which were determined prior to the formation of the current consortium). The scores for symptoms included neuropathy symptom score (NSS), neuropathy symptom profile (NSP), and diabetic neuropathy symptom (DNS) score and the scores for signs included the neuropathy disability score (NDS) and Toronto clinical neuropathy score (TCNS). The algorithm for the reference standard was positive if two criteria were met: 1) if a clinical symptom or clinical signs were present and 2) if peroneal motor nerve conduction velocity was less than 42 m/s. This reference standard was based on consensus criteria.[13-15]

Reference standard definitions using other combinations of abnormal peroneal and/or sural nerve parameters and/or presence of signs and symptoms were considered in sensitivity analysis. Other nerve conduction parameters included sural nerve amplitude potential and conduction

velocity, and peroneal nerve amplitude potential, conduction velocity, and F-wave latency (adjusted for height). Amplitude potentials were adjusted for age. Specifically, these alternate reference standards included the following 6 case-definitions: i) abnormal sural nerve amplitude potential (≤7.2 μV if age ≤65 and ≤5.5 μV if age >65); ii) using each centre's local case-definition where nerve conduction values were considered abnormal when greater than the 99th percentile or less than the 1st percentile in the reference healthy population database used at each site, according to the ranges determined during certification of the clinical laboratories; iii) any abnormal sural nerve parameter, corroborated by any abnormal peroneal parameter, corroborated by the presence of at least one sign or one symptom ("Toronto site's definition"); iv) the study's reference standard described above; v) the presence of a high number of signs and/or symptoms ("Clinical Definition"); and vi) the presence of a high number of signs and/or symptoms, corroborated by peroneal motor nerve conduction velocity <42 m/s ("Stringent Clinical Definition").

Variables Used for Sensitivity Analyses

Sensitivity analyses were undertaken to account for an imperfect reference standard.[14] Specifically, electrophysiology and clinically relevant signs and symptoms detect large nerve fibre dysfunction and may fail to identify patients with early small fibre neuropathy detected using IVCCM.[16] Skin Biopsy for IENFD assessment was undertaken in a subset of the Manchester participants,[17] and was used in resolver test analysis (Sensitivity Analysis #2). Other evaluations included blood pressure, smoking history and biochemical tests including glycated hemoglobin A_{1c}, serum lipids, and urinary albumin excretion (generally conducted on the same day as or within 1 week of the neuropathy evaluation). Cooling detection thresholds were determined in the majority of participants by CASE IV (WR Medical Electronics Co., MN,

USA) or the Medoc TSA-II NeuroSensory Analyzer (Medoc Advanced Medical Systems, Ramat-Yishai, Israel), using the method of limits.

Statistics

The available sample size yielded a power of >0.99 to detect a conservative area under the receiver operating characteristic curve (AUC) of 0.70 from the null hypothesis of 0.50. AUC was compared within study populations using the method of Pencina et al.[18] AUC was compared between study populations using the method of Hanley & McNeil.[19] Optimal diagnostic thresholds were identified by distance to the point of perfect discrimination using the formula $\sqrt{(0-x)^2+(1-y)^2}$. The sensitivity analyses to account for imperfect reference standard included: 1) modification of the reference standard definition parameters to create less- and more-stringent definitions (as described above); 2) use of composite reference standard methods incorporating small fibre measures of intra-epidermal nerve fibre density into the definition of neuropathy cases;[17] and 3) latent class analysis that identified clusters of patients who shared common clinical characteristics and neurological test results (including the clinical scales, cooling detection threshold tests,[20] and electrophysiological tests) that were consistent with the presence of neuropathy.[21] Details of variables used in these analyses are provided above. ROC regression was performed according to the method of Janes, Longton, and Pepe.[22] Additionally, while we focused on the optimal diagnostic threshold as the single value that simultaneously maximized sensitivity and specificity, we conducted an alternate approach in which a pair of diagnostic thresholds were determined - one lower value chosen to maximize specificity (more confidently rule in the presence of neuropathy) and one higher value chosen to simultaneously maximize sensitivity (more confidently rule out the presence of neuropathy). To accomplish this, we used a combination of decision criteria that included threshold values that i)

maximized positive and negative likelihood ratios, and ii) minimized false positives and false negatives.

Section 2. ESM Figures and Tables

2. ESM Tables

ESM Table 1. Characteristics of the 998 study participants, according to study centre.

Characteristic	Total N=998	Brisbane n=235	Calgary n=84	Manchester n=281	Michigan n=17	Toronto n=381	*p*-value
Female Sex	420 (42%)	110 (47%)	40 (48%)	110 (39%)	5 (29%)	155 (41%)	0.23
Age (y)	52±18	52 ± 14	15 ± 2	58 ± 14	51 ± 13	55 ± 17	<0.001
Ethnicity							
Aboriginal North American	1 (0%)	0 (0%)	1 (1%)	0 (0%)	0 (0%)	0 (0%)	
Asian	132 (13%)	11 (5%)	5 (6%)	81 (29%)	0 (0%)	35 (9%)	
Black	11 (1%)	0 (0%)	1 (1%)	3 (1%)	1 (6%)	6 (2%)	
Hawaiian or Pacific Islander	1 (0%)	1 (0%)	0 (0%)	0 (0%)	0 (0%)	0 (0%)	
Hispanic	15 (2%)	0 (0%)	1 (1%)	0 (0%)	0 (0%)	14 (4%)	
Middle Eastern	5 (1%)	3 (1%)	0 (0%)	2 (1%)	0 (0%)	0 (0%)	
White	799 (80%)	209 (89%)	75 (89%)	195 (69%)	13 (76%)	307 (81%)	
Other/Unknown/Unreported	34 (3%)	11 (5%)	1 (1%)	0 (0%)	3 (18%)	19 (5%)	
Diabetes duration (y)	17±13	18 ± 14	9 ± 3	21 ± 14	9 ± 4	16 ± 13	<0.001
BMI (kg/m^2)	28.1±6.1	28.8 ± 6.2	21.6 ± 3.2	29.0 ± 5.9	33.8 ± 5.8	28.3 ± 5.5	<0.001
A_{1c} (mmol/mol)	63±18	63 ± 16	74 ± 19	64 ± 17	66 ± 13	60 ± 18	<0.001
A_{1c} (%)	7.9±1.6	7.9 ± 1.5	8.9 ± 1.8	8.0 ± 1.6	8.2 ± 1.2	7.6 ± 1.6	<0.001
Neurological exam							
Sign Score Used	-	NDS	NDS	NDS	-	TCNS	-
Median Sign Score	-	1[0,3]	0[0,0]	3[1,6]	-	6[3,9]	-
Sign(s) Present	721 (72%)	138 (59%)	14 (17%)	217 (78%)	15 (88%)	337 (90%)	<0.001
Symptom Score Used	-	DNS	NSS	NSP	-	TCNS	-
Median Symptom Score	-	0[0,1]	0[0,0]	3[1,7]	-	2[0,4]	-
Symptom(s) Present	606 (61%)	90 (38%)	2 (2%)	222 (80%)	14 (82%)	278 (73%)	<0.001
Nerve conduction studies							
Sural AMP (µV)	8.3 ± 7.9	8.2 ± 9.4	17.9 ± 7.1	9.1 ± 7.2	5.2 ± 5.9	5.8 ± 5.6	<0.001
Sural CV (m/s)	41.2 ± 7.1	37.4 ± 6.7	45.9 ± 4.9	43.1 ± 7.6	39.8 ± 5.4	41.3 ± 6.4	<0.001
Peroneal AMP (mV)	3.7 ± 2.6	4.2 ± 2.7	5.3 ± 1.8	3.2 ± 2.6	-	3.5 ± 2.6	<0.001
Peroneal CV (m/s)	41.4 ± 7.5	43.6 ± 7.3	46.9 ± 4.5	41.4 ± 8.0	37.7 ± 7.6	39.0 ± 6.7	<0.001
Peroneal F-wave (ms)	57.9 ± 10.3	56.0 ± 7.7	-	55.4 ± 8.9	-	60.9 ± 11.8	<0.001
DSP present	415 (42%)	57 (24%)	0 (0%)	120 (43%)	12 (71%)	226 (59%)	<0.001

IVCCM Parameters

Automated protocol

Section 2. ESM Figures and Tables

Characteristic	Total N=998	Brisbane n=235	Calgary n=84	Manchester n=281	Michigan n=17	Toronto n=381	*p*-value
$CNFL_{AUTO}$ (mm/mm^2)	12.5 ± 4.6	15.6 ± 3.9	15.2 ± 2.9	12.2 ± 4.6	15.3 ± 6.4	10.2 ± 3.6	<0.001
$CNBD_{AUTO}$ ($branches/mm^2$)	22.7 ± 18.3	24.8 ± 19.7	30.6 ± 13.6	25.3 ± 19.4	-	17.9 ± 16.2	<0.001
$CNFD_{AUTO}$ ($fibres/mm^2$)	20.6 ± 9.8	17.3 ± 7.4	24.5 ± 5.8	19.0 ± 8.9	-	22.9 ± 11.4	<0.001
Manual protocol							
$CNFL_{MANUAL}$ (mm/mm^2)	17.3 ± 6.5	17.8 ± 6.5	22.8 ± 4.7	19.2 ± 7.3	-	14.5 ± 4.7	<0.001
$CNBD_{MANUAL}$ ($branches/mm^2$)	50.9 ± 40.0	68.6 ± 52.5	70.1 ± 30.1	55.9 ± 37.9	-	32.4 ± 23.5	<0.001
$CNFD_{MANUAL}$ ($fibres/mm^2$)	38.6 ± 26.3	98.6 ± 44.8	31.3 ± 7.4	24.3 ± 8.7	-	38.3 ± 11.7	<0.001
T1DM/T2DM	516 / 482	156 / 79	84 / 0	135 / 146	2 / 15	139 / 242	<0.001

NDS, neuropathy disability score; TCNS, Toronto clinical neuropathy score; DNS, diabetic neuropathy symptom; DSP, diabetic sensorimotor polyneuropathy; NSS, neuropathy symptom score; NSP, neuropathy symptom profile; IVCCM, in vivo corneal confocal microscopy; CNFL, corneal nerve fibre length; CNBD, corneal nerve branch density; CNFD, corneal nerve fibre density. T1DM, type 1 diabetes mellitus; T2D, type 2 diabetes mellitus. P-value from test for trend.

ESM Table 2. Selected neurological characteristics and mean IVCCM parameters in the derivation sets, according to the absence or presence of DSP.

Parameter	Neuropathy Controls	Neuropathy Cases	*p*-value
**Type 1 diabetes**	182 (70%)	78 (30%)	
Neurological exam			
Sign(s) present	76 (42%)	72 (94%)	<0.001
Symptom(s) present	37 (20%)	68 (87%)	<0.001
Nerve conduction studies			
Sural AMP (µV)	13.0 ± 8.2	3.3 ± 4.4	<0.001
Peroneal CV (m/s)	45.3 ± 4.7	34.3 ± 7.1	<0.001
IVCCM parameters			
$CNFL_{AUTO}$ (mm/mm^2)	14.1 ± 3.9	9.6 ± 4.5	<0.001
$CNBD_{AUTO}$ ($branches/mm^2$)	25.4 ± 16.1	13.9 ± 14.0	<0.001
$CNFD_{AUTO}$ ($fibres/mm^2$)	21.9 ± 8.4	15.2 ± 9.6	<0.001
$CNFL_{MANUAL}$ (mm/mm^2)	19.4 ± 5.7	13.7 ± 6.1	<0.001
$CNBD_{MANUAL}$ ($branches/mm^2$)	58.4 ± 34.4	35.3 ± 30.4	<0.001
$CNFD_{MANUAL}$ ($fibres/mm^2$)	34.1 ± 10.3	25.3 ± 12.3	<0.001
**Type 2 diabetes**	115 (49%)	121 (51%)	
Neurological exam			
Sign(s) present	86 (77%)	110 (98%)	<0.001
Symptom(s) present	71 (63%)	112 (97%)	<0.001
Nerve conduction studies			
Sural AMP (µV)	9.6 ± 7.4	3.1 ± 4.2	<0.001
Peroneal CV (m/s)	46.3 ± 5.1	35.5 ± 5.6	<0.001
IVCCM parameters			
$CNFL_{AUTO}$ (mm/mm^2)	13.6 ± 4.3	11.0 ± 4.0	<0.001
$CNBD_{AUTO}$ ($branches/mm^2$)	29.5 ± 20.0	19.7 ± 18.0	<0.001
$CNFD_{AUTO}$ ($fibres/mm^2$)	21.9 ± 9.4	21.3 ± 11.4	0.63
$CNFL_{MANUAL}$ (mm/mm^2)	19.4 ± 6.9	15.0 ± 5.8	<0.001
$CNBD_{MANUAL}$ ($branches/mm^2$)	67.7 ± 49.5	39.5 ± 34.8	<0.001
$CNFD_{MANUAL}$ ($fibres/mm^2$)	51.3 ± 40.4	37.3 ± 20.7	0.28
**Total Derivation Set**	297 (60%)	199 (40%)	
Neurological exam			
Sign(s) present	162 (55%)	182 (96%)	<0.001
Symptom(s) present	108 (37%)	180 (93%)	<0.001
Nerve conduction studies			
Sural AMP (µV)	11.7 ± 8.1	3.2 ± 4.2	<0.001
Peroneal CV (m/s)	45.7 ± 4.9	35.0 ± 6.2	<0.001
IVCCM parameters			
$CNFL_{AUTO}$ (mm/mm^2)	13.9 ± 4.1	10.5 ± 4.2	<0.001
$CNBD_{AUTO}$ ($branches/mm^2$)	27.0 ± 17.8	17.4 ± 17.4	<0.001
$CNFD_{AUTO}$ ($fibres/mm^2$)	21.9 ± 8.8	18.8 ± 11.1	0.001
$CNFL_{MANUAL}$ (mm/mm^2)	19.4 ± 6.2	14.5 ± 6.0	<0.001
$CNBD_{MANUAL}$ ($branches/mm^2$)	62.0 ± 41.1	37.9 ± 33.1	<0.001
$CNFD_{MANUAL}$ ($fibres/mm^2$)	42.6 ± 30.4	33.1 ± 19.1	0.012

AMP, amplitude potential; DSP, diabetic sensorimotor polyneuropathy; CV, conduction velocity; IVCCM, in vivo corneal confocal microscopy; CNFL, corneal nerve fibre length; CNBD, corneal nerve branch density; CNFD, corneal nerve fibre density.

ESM Table 3. Characteristics and comparisons of ROC curves for DSP in the derivation and validation sets, and in the total study population.

				Optimal Thresholds		
Parameter	AUC	95% CI for AUC	*p*-value	Value	Sn	Sp
**Derivation Sets**						
Type 1 diabetes						
$CNFL_{AUTO}$	0.77	0.71, 0.84	-	<12.5 mm/mm^2	0.73	0.69
$CNBD_{AUTO}$	0.73	0.66, 0.80	<0.001	<12.5 branches/mm^2	0.62	0.78
$CNFD_{AUTO}$	0.71	0.63, 0.78	<0.001	<16.5 fibres/mm^2	0.56	0.71
$CNFL_{MANUAL}$	0.75	0.69, 0.82	0.090	<16.4 mm/mm^2	0.71	0.67
$CNBD_{MANUAL}$	0.72	0.65, 0.79	<0.001	<37.6 branches/mm^2	0.67	0.72
$CNFD_{MANUAL}$	0.70	0.62, 0.79	0.001	<28.0 fibres/mm^2	0.65	0.75
Type 2 diabetes						
$CNFL_{AUTO}$	0.68	0.62, 0.75	-	<12.3 mm/mm^2	0.69	0.63
$CNBD_{AUTO}$	0.66	0.59, 0.73	0.011	<18.8 branches/mm^2	0.62	0.67
$CNFD_{AUTO}$	0.52	0.44, 0.59	<0.001	<22.8 fibres/mm^2	0.59	0.48
$CNFL_{MANUAL}$	0.69	0.63, 0.76	0.13	<16.3 mm/mm^2	0.65	0.69
$CNBD_{MANUAL}$	0.69	0.62, 0.76	0.18	<44.8 branches/mm^2	0.69	0.63
$CNFD_{MANUAL}$	0.54	0.47, 0.62	0.012	<39.2 fibres/mm^2	0.69	0.41
**Validation Sets**						
Type 1 diabetes						
$CNFL_{AUTO}$	0.74	0.68, 0.81	-	<11.7 mm/mm^2	0.67	0.74
$CNBD_{AUTO}$	0.65	0.58, 0.72	<0.001	<18.8 branches/mm^2	0.67	0.57
$CNFD_{AUTO}$	0.69	0.61, 0.76	<0.001	<17.9 fibres/mm^2	0.67	0.68
$CNFL_{MANUAL}$	0.69	0.62, 0.76	<0.001	<16.7 mm/mm^2	0.73	0.64
$CNBD_{MANUAL}$	0.66	0.58, 0.73	<0.001	<40.2 branches/mm^2	0.70	0.57
$CNFD_{MANUAL}$	0.58	0.48, 0.67	<0.001	<28.0 fibres/mm^2	0.48	0.70
Type 2 diabetes						
$CNFL_{AUTO}$	0.63	0.56, 0.70	-	<12.2 mm/mm^2	0.63	0.57
$CNBD_{AUTO}$	0.61	0.53, 0.68	0.15	<19.5 branches/mm^2	0.65	0.62
$CNFD_{AUTO}$	0.54	0.47, 0.61	<0.001	<16.8 fibres/mm^2	0.49	0.65
$CNFL_{MANUAL}$	0.65	0.58, 0.72	0.044	<16.3 mm/mm^2	0.66	0.63
$CNBD_{MANUAL}$	0.62	0.54, 0.69	0.32	<44.5 branches/mm^2	0.67	0.55
$CNFD_{MANUAL}$	0.52	0.45, 0.60	<0.001	<35.9 fibres/mm^2	0.58	0.47
**Total Study Population (N=998)**						
$CNFL_{AUTO}$	0.71	0.68, 0.74	-	<12.3 mm/mm^2	0.67	0.66
$CNBD_{AUTO}$	0.65	0.61, 0.69	<0.001	<18.7 branches/mm^2	0.66	0.60
$CNFD_{AUTO}$	0.60	0.56, 0.64	<0.001	<16.7fibres/mm^2	0.52	0.68
$CNFL_{MANUAL}$	0.70	0.66, 0.73	0.006	<16.3 mm/mm^2	0.67	0.66
$CNBD_{MANUAL}$	0.67	0.63, 0.70	<0.001	<38.9 branches/mm^2	0.64	0.63
$CNFD_{MANUAL}$	0.55	0.51, 0.59	<0.001	<27.8 fibres/mm^2	0.41	0.70

P-value for comparison of AUC with that of $CNFL_{AUTO}$ (within the corresponding derivation set, validation set, or total study population).

Sn, sensitivity; Sp, specificity; CNFL, corneal nerve fibre length; CNBD, corneal nerve branch density; CNFD, corneal nerve fibre density.

ESM Table 4. Optimal threshold values and alternate pairs of thresholds for CNFL – with lower values meant to maximize specificity and higher values meant to maximize sensitivity – and their operating characteristics for identifying DSP in the total study population and the derivation and validation sets for type 1 and type 2 diabetes.

	Optimal Single Threshold*	Lower Threshold of the Threshold Pair				Higher Threshold of the Threshold Pair				Classification Scheme Using Threshold Pair				
											Misclassified			
	Value	Value	Sp	PPV	LR+	Value	Sn	NPV	LR-	Correct	Total	FP	FN	Unclassified
**Total Study Population**														
CNFL$_{AUTO}$	<12.3	<8.6	0.88	0.66	2.7	<15.3	0.88	0.81	0.33	35.2%	12.2%	7.1%	5.1%	52.6%
CNFL$_{MANUAL}$	<16.3	<10.91	0.88	0.64	2.6	<21.8	0.89	0.81	0.33	31.0%	11.2%	6.9%	4.3%	57.8%
**Type 1 diabetes derivation set**														
CNFL$_{AUTO}$	<12.5	<8.8	0.90	0.65	4.5	<15.0	0.88	0.90	0.27	43.1%	10.8%	7.3%	3.5%	46.2%
CNFL$_{MANUAL}$	<16.4	<12.1	0.88	0.63	4.5	<21.8	0.88	0.88	0.33	37.8%	11.5%	8.1%	3.5%	50.8%
**Type 2 diabetes derivation set**														
CNFL$_{AUTO}$	<12.3	<8.7	0.90	0.74	2.9	<15.0	0.88	0.75	0.31	33.1%	11.4%	5.1%	6.4%	55.5%
CNFL$_{MANUAL}$	<16.3	<12.1	0.83	0.67	2.0	<21.3	0.90	0.79	0.27	35.6%	13.6%	8.1%	5.5%	50.9%
**Derived thresholds applied to the validation sets**														
Type 1 diabetes														
CNFL$_{AUTO}$	<12.5	<8.8	0.87	0.56	2.7	<15.0	0.88	0.88	0.29	40.2%	12.9%	9.0%	3.9%	46.9%
CNFL$_{MANUAL}$	<16.4	<12.1	0.85	0.55	2.6	<21.8	0.90	0.85	0.36	30.9%	13.3%	10.2%	3.1%	55.9%
Type 2 diabetes														
CNFL$_{AUTO}$	<12.3	<8.7	0.80	0.66	1.6	<15.0	0.84	0.63	0.48	32.5%	17.8%	8.9%	8.9%	49.6%
CNFL$_{MANUAL}$	<16.3	<12.1	0.78	0.63	1.4	<21.3	0.87	0.67	0.41	32.1%	17.5%	10.2%	7.3%	50.4%

Values are given in mm/mm^2. DSP, diabetic sensorimotor polyneuropathy; Sn, sensitivity; Sp, specificity; PPV, positive predictive value; LR+, positive likelihood ratio; NPV, negative predictive value; LR-, negative likelihood ratio; FP, false positives; FN, false negatives.

*Optimal single threshold from the primary analysis shown for comparison. This single threshold optimized sensitivity and specificity simultaneously. The pair of thresholds was used to determine an interval that optimized sensitivity and specificity individually. For example, for CNFL$_{AUTO}$ in the total study population, the interval values chosen were 8.6 and 15.3 mm/mm^2. Values <8.3 mm/mm^2 rule in neuropathy, values ≥15.3 mm/mm^2 rule out neuropathy, and values within the interval 8.6-15.3 mm/mm^2 represent unclassified participants.

ESM Table 5. Details of Sensitivity Analysis #2 and Sensitivity Analysis #3 (undertaken to address use of a possible imperfect reference standard).

				Study Reference Standard			Alternate definition			
Sensitivity Analysis Number	Alternate method to define cases	DM Type	IVCCM parameter	N cases	N controls	AUC (95% CI)	N cases	N controls	AUC (95% CI)	*p*-value
2	Composite reference standard	T1DM	$CNFL_{AUTO}$	41	43	0.73 (0.62, 0.84)	41	43	0.77 (0.67, 0.88)	0.58
		T1DM	$CNFL_{MANUAL}$	41	43	0.67 (0.55, 0.79)	41	43	0.70 (0.58, 0.82)	0.75
		T2DM	$CNFL_{AUTO}$	17	33	0.67 (0.52, 0.83)	27	23	0.51 (0.35, 0.68)	0.16
		T2DM	$CNFL_{MANUAL}$	17	33	0.70 (0.55, 0.85)	27	23	0.50 (0.34, 0.67)	0.08
3	Latent Class Analysis	T1DM	$CNFL_{AUTO}$	151	342	0.76 (0.71, 0.81)	76	417	0.80 (0.75, 0.85)	0.28
		T1DM	$CNFL_{MANUAL}$	151	342	0.72 (0.67, 0.77)	76	417	0.73 (0.66, 0.79)	0.81
		T2DM	$CNFL_{AUTO}$	214	215	0.66 (0.60, 0.71)	127	302	0.68 (0.63, 0.73)	0.52
		T2DM	$CNFL_{MANUAL}$	214	215	0.67 (0.62, 0.72)	127	302	0.69 (0.64, 0.75)	0.60

Due to differences in case-composition between definitions for DSP, comparisons of AUC were made using the method of Hanley and McNeil[19].

CNFL, corneal nerve fibre length; DM, diabetes mellitus; T1DM, type 1 diabetes mellitus; T2DM, type 2 diabetes mellitus.

3. ESM Figures

ESM Fig. 1. Flow of participants.

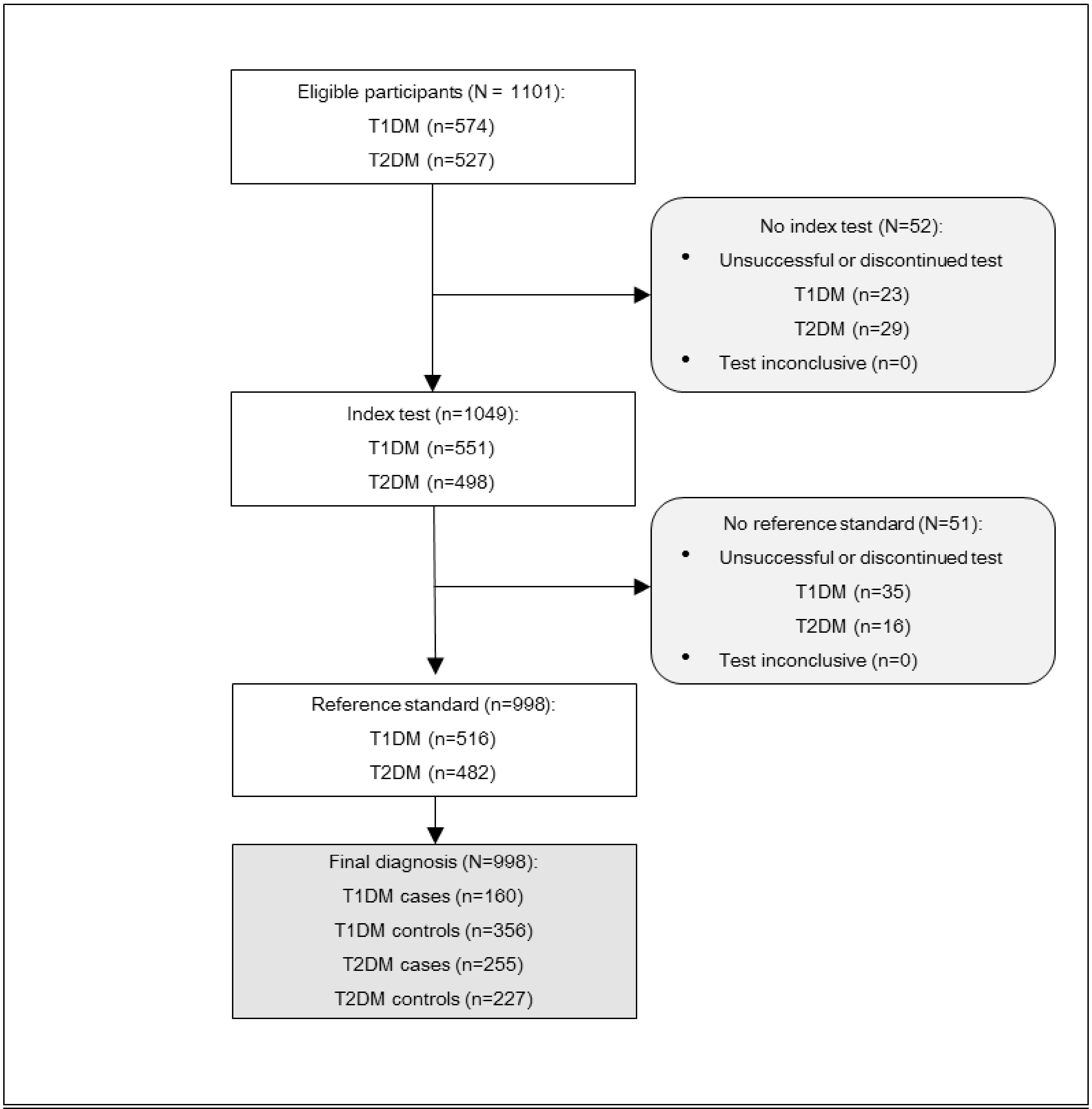

ESM Fig. 1 Legend: Index test was IVCCM and reference standard was clinical and electrophysiological assessment. T1DM, type 1 diabetes participants; T2DM, type 2 diabetes participants.

Section 3. ESM Figures and Tables

ESM Fig. 2. AUC for CNFL when altering the reference standard definitions for DSP.

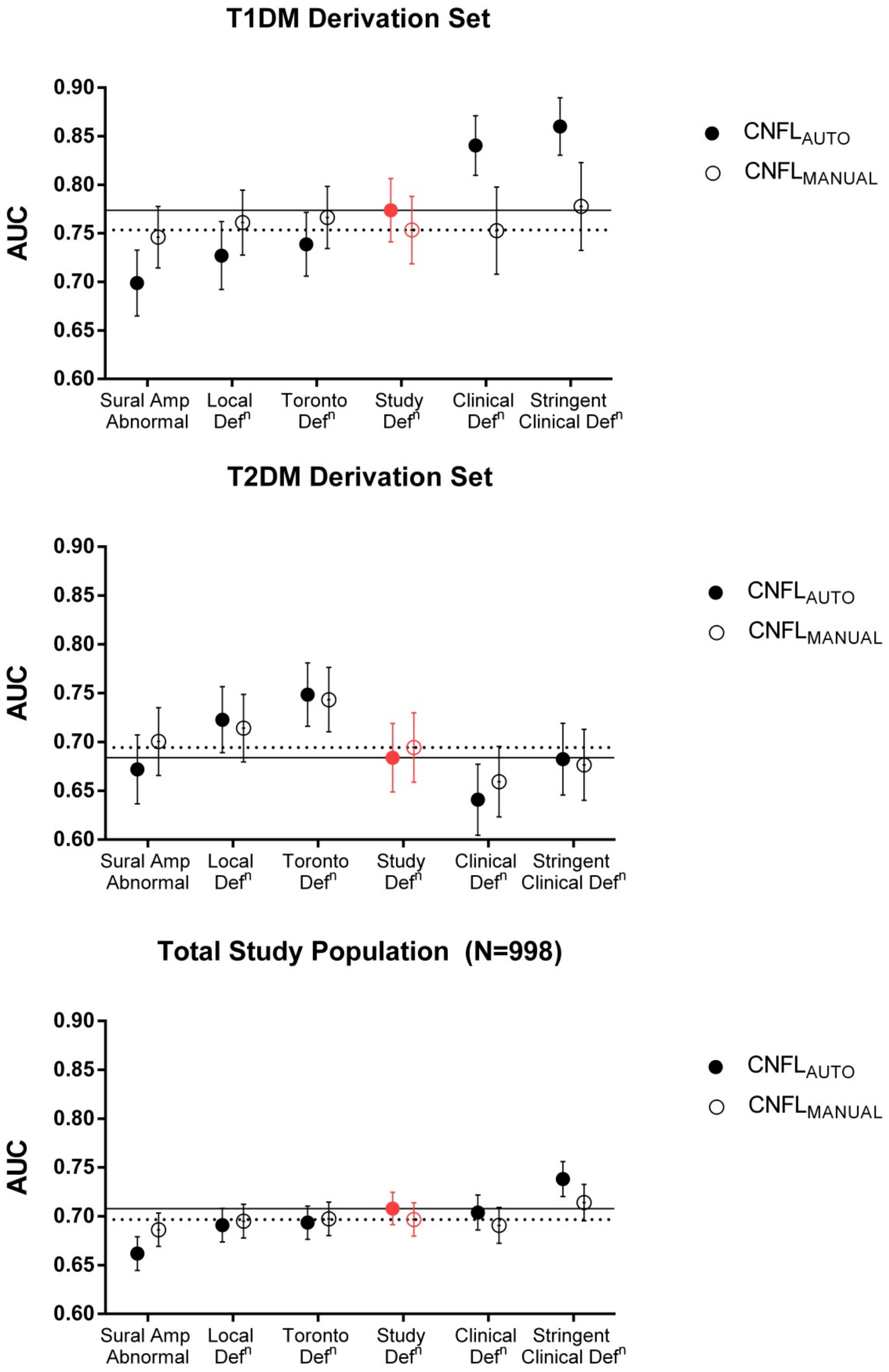

Section 3. ESM Figures and Tables

ESM Fig. 2 Legend: Definitions to the left of the study definition represent less stringent definitions of neuropathy, while definitions to the right represent more stringent definitions. Red symbols indicate AUC for the study's reference standard definition. The solid horizontal lines represent the AUC for $CNFL_{AUTO}$ using the study definition as the reference standard. The dashed horizontal lines represent the AUC for $CNFL_{MANUAL}$ using the study definition as the reference standard. Error bars represent the standard error for AUC.
CNFL, corneal nerve fibre length; DSP, diabetic sensorimotor polyneuropathy; T1DM, type 1 diabetes mellitus; T2DM, type 2 diabetes mellitus; Def^{n}, definition.

Section 4. ESM References.

ESM References

[1] Pacaud D, Romanchuk KG, Tavakoli M, et al. (2015) The Reliability and Reproducibility of Corneal Confocal Microscopy in Children. Invest Ophthalmol Vis Sci 56: 5636-5640

[2] Stem MS, Hussain M, Lentz SI, et al. (2014) Differential reduction in corneal nerve fiber length in patients with type 1 or type 2 diabetes mellitus. J Diabetes Complications 28: 658-661

[3] Tavakoli M, Quattrini C, Abbott C, et al. (2010) Corneal confocal microscopy: a novel noninvasive test to diagnose and stratify the severity of human diabetic neuropathy. Diabetes Care 33: 1792-1797

[4] Ahmed A, Bril V, Orszag A, et al. (2012) Detection of diabetic sensorimotor polyneuropathy by corneal confocal microscopy in type 1 diabetes: a concurrent validity study. Diabetes Care 35: 821-828

[5] Edwards K, Pritchard N, Vagenas D, Russell A, Malik RA, Efron N (2012) Utility of corneal confocal microscopy for assessing mild diabetic neuropathy: baseline findings of the LANDMark study. Clin Exp Optom 95: 348-354

[6] Tavakoli M, Malik RA (2011) Corneal confocal microscopy: a novel non-invasive technique to quantify small fibre pathology in peripheral neuropathies. J Vis Exp 47: 2194-3001

[7] Hume DA, Lovblom LE, Ahmed A, et al. (2012) Higher magnification lenses versus conventional lenses for evaluation of diabetic neuropathy by corneal in vivo confocal microscopy. Diabetes Res Clin Pract 97: e37-40

[8] Scarr D, Lovblom LE, Ostrovski I, et al. (2017) Agreement between automated and manual quantification of corneal nerve fiber length: Implications for diabetic neuropathy research. J Diabetes Complications 31: 1066-1073

[9] Chen X, Graham J, Dabbah MA, Petropoulos IN, Tavakoli M, Malik RA (2017) An Automatic Tool for Quantification of Nerve Fibers in Corneal Confocal Microscopy Images. IEEE Trans Biomed Eng 64: 786-794

[10] Halpern EM, Lovblom LE, Orlov S, Ahmed A, Bril V, Perkins BA (2013) The impact of common variation in the definition of diabetic sensorimotor polyneuropathy on the validity of corneal in vivo confocal microscopy in patients with type 1 diabetes: a brief report. J Diabetes Complications 27: 240-242

[11] Ostrovski I, Lovblom LE, Farooqi MA, et al. (2015) Reproducibility of In Vivo Corneal Confocal Microscopy Using an Automated Analysis Program for Detection of Diabetic Sensorimotor Polyneuropathy. PLoS One 10: e0142309

Section 4. ESM References.

[12] Chen X, Graham J, Dabbah MA, et al. (2015) Small nerve fiber quantification in the diagnosis of diabetic sensorimotor polyneuropathy: comparing corneal confocal microscopy with intraepidermal nerve fiber density. Diabetes Care 38: 1138-1144

[13] England JD, Gronseth GS, Franklin G, et al. (2005) Distal symmetric polyneuropathy: a definition for clinical research: report of the American Academy of Neurology, the American Association of Electrodiagnostic Medicine, and the American Academy of Physical Medicine and Rehabilitation. Neurology 64: 199-207

[14] Tesfaye S, Boulton AJ, Dyck PJ, et al. (2010) Diabetic neuropathies: update on definitions, diagnostic criteria, estimation of severity, and treatments. Diabetes Care 33: 2285-2293

[15] Pop-Busui R, Boulton AJ, Feldman EL, et al. (2017) Diabetic Neuropathy: A Position Statement by the American Diabetes Association. Diabetes Care 40: 136-154

[16] Bril V (1994) Role of electrophysiological studies in diabetic neuropathy. Can J Neurol Sci 21: S8-12

[17] Quattrini C, Tavakoli M, Jeziorska M, et al. (2007) Surrogate markers of small fiber damage in human diabetic neuropathy. Diabetes 56: 2148-2154

[18] Pencina MJ, D'Agostino RB, Sr., D'Agostino RB, Jr., Vasan RS (2008) Evaluating the added predictive ability of a new marker: from area under the ROC curve to reclassification and beyond. Stat Med 27: 157-172; discussion 207-112

[19] Hanley JA, McNeil BJ (1983) A method of comparing the areas under receiver operating characteristic curves derived from the same cases. Radiology 148: 839-843

[20] Levy D, Abraham R, Reid G (1989) A comparison of two methods for measuring thermal thresholds in diabetic neuropathy. J Neurol Neurosurg Psychiatry 52: 1072-1077

[21] Rutjes AW, Reitsma JB, Coomarasamy A, Khan KS, Bossuyt PM (2007) Evaluation of diagnostic tests when there is no gold standard. A review of methods. Health Technol Assess 11: iii, ix-51

[22] Janes H, Longton G, Pepe M (2009) Accommodating Covariates in ROC Analysis. Stata J 9: 17-39
